# Ophthalmic Presentations and Manifestations of COVID-19: A Systematic Review of Global Observations

**DOI:** 10.7759/cureus.40695

**Published:** 2023-06-20

**Authors:** Jordan L Pace, Drew Richard, Adon Khachik, Mehul Mistry, Gagandeep Singh, Navid Mostaghni, Susan Yazdanmehr

**Affiliations:** 1 Medicine, California University of Science and Medicine, Colton, USA; 2 Medical Education, California University of Science and Medicine, Colton, USA

**Keywords:** complications, covid-19, ophthalmic, infectious disease, microbiology

## Abstract

As the presentations and complications of severe acute respiratory syndrome coronavirus 2 (SARS-CoV-2) continue to surface, the ocular manifestations have emerged as an area of interest. Research and reports conveyed the presence of several ophthalmic conditions observed in Coronavirus disease 2019 (COVID-19) patients. These publications documented a range of presentations varying from asymptomatic to serious impairments. The aim of this study is to characterize the ophthalmic pathologies and their frequencies observed due to COVID-19 in patients across different regions of the world. The goal is that the paper assists primary care physicians and healthcare providers.

A systematic review of 31 articles published between January 1, 2021 to January 13, 2022, explored the presenting ocular symptoms of COVID-19, diagnosis, duration of ophthalmic complications, as well as pre-existing comorbidities. A total of 816 patients, 427 (52.3%) males and 389 (47.7%) females, from various regions of the world were investigated. Studies focusing on patients with a history of ocular pathologies, non-COVID-19 infections, complications associated with the COVID-19 vaccine, and pediatric patients were excluded from this study.

Ocular complications were most commonly reported one to two weeks following the initial COVID-19 diagnosis. Analysis suggests that the “red” eye is the most prevalent presenting ophthalmologic symptom, followed by temporary vision loss. Conjunctivitis was also the most common clinical diagnosis reported, followed by neuro-retinal affection in the form of cotton wool spots (n=127 and n=9, respectively).

This study summarizes ocular manifestations in COVID-19 patients and serves to help healthcare providers recognize common symptoms and their severity. This may lead to early diagnosis, treatment, and intervention of these manifestations.

## Introduction and background

References [1-31] were used for data extraction. References [32-49] were cited throughout the manuscript. Entering 2022, the world embarks on its third year since the SARS-CoV-2 pandemic outbreak began in Wuhan, China, at the end of 2019 [31]. Nearing the end of 2021, there were nearly 300 million documented cases of COVID-19 infection, with approximately 5.5 million deaths reported [32]. The concern for complications following COVID-19 infection gives researchers cause for investigating COVID-19 survivors. “Long-COVID” has been the term developed to explain the health challenges experienced by individuals, sometimes even after recovering from the viral infection [33,39].

While the pathological effects of COVID-19 were initially emphasized on the respiratory system, other organ systems are beginning to receive added attention. In particular, the eyes have become a particular organ of study. Initial research found that COVID-19 could be transmitted via the lacrimal ducts of the eye [34]. SARS-CoV-2 was found to bind angiotensin-converting enzyme 2 (ACE2) receptor and transmembrane protease serine 2 (TMPRSS2). These receptors also exist in the tissue of the eyes and represent a possible route of entry via the eyes [35-37]. Beyond a potential vector for transmission, the eyes have also expressed an area of research for the manifestations and complications of this disease. Initial documentation has reported various symptoms from dry eye, foreign body sensation, itching, conjunctivitis, and visual acuity changes [38]. Most cases reported were transient and ocular abnormalities resolved following COVID-19 recovery.

Despite the increasing attention, there remains an incomplete understanding of the ocular manifestations and complications surrounding COVID-19 infection. Challenges are exacerbated by the unknown prevalence of asymptomatic COVID-19 carriers that are unaware and not receiving medical care [40]. As case reports, case series and larger-scale research are being conducted and published, we can trend more cases and observe longer-term ocular consequences of COVID-19 infection. Understanding the ophthalmological signs and sequelae of COVID-19 infection can help healthcare workers better triage their patients and deliver quality care.

This paper intends to update the current literature on ocular manifestations and their relationship with COVID-19 infection. We hope to illuminate a clearer view of considerations to be made when encountering patients within and outside of an ocular setting amid the COVID-19 pandemic. This review supplies a comprehensive breakdown of the data collected regarding COVID-19 and ocular symptoms experienced by patients worldwide.

This manuscript is currently online by Preprint SSRN of Lancet as of September 22, 2022.

## Review

Methods

Literature Search Criteria

To identify eligible studies, a literature search was performed in PubMed for accessible articles with publication dates between January 1, 2021 and January 13, 2022. The following search terms were applied to identify publications available in English: “COVID-19” AND “ophthalmologic” OR “OPTIC” AND “anterior segment” OR “conjunctiva” OR “ocular surface” OR “retina” OR “retinitis” OR “retinal vein occlusion” OR “glaucoma” OR “conjunctivitis” OR “choroid” OR “uveitis” OR “neuro-ophthalmology” OR “cranial nerve palsy” OR “orbit” AND “manifestations” OR “complications.”

**Figure 1:**
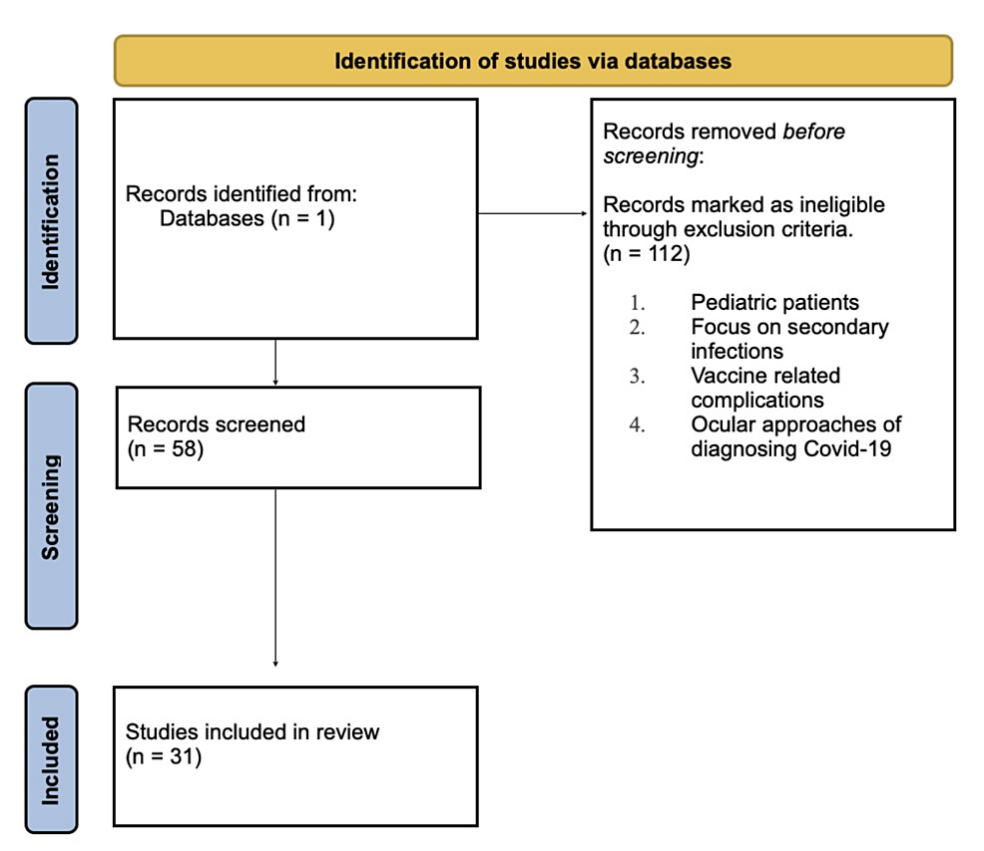
Preferred Reporting Items for Systematic Reviews and Meta-Analyses (PRISMA) flow chart followed for inclusion and exclusion criteria

The initial search resulted in 170 studies using one database (PubMed) (Figure 1). Abstracts of the accessible studies were independently reviewed by two reviewers for eligibility per inclusion and exclusion criteria. Articles were selected for the final analysis if they observed any relationship between COVID-19 infection and ophthalmic manifestations, and infection was confirmed through positive diagnostic tests indicating either previous COVID-19 infection by antibody assay or active infection confirmed by viral reverse transcription-polymerase chain reaction testing (RT-PCR). Exclusion criteria included: studies on pediatric patients younger than 18 years of age, studies with a primary focus on ocular complications due to secondary infection such as mucor mycosis, herpes simplex virus (HSV), and cytomegalovirus (CMV), COVID-19 vaccine-related complications, and papers that focused on the viral-load detection of COVID-19 in conjunctival surfaces.

After applying the inclusion and exclusion criteria, 31 accessible papers were finalized to be included in this review [1-31]. The 31 selected studies had a total of 816 subjects. Publications included 27 case reports, a case series, a cross-sectional study, and a cross-sectional cohort study.

Literature Review Process and Data Collection

Six reviewers extracted information from 31 publications. Each paper was initially examined by two independent reviewers and discrepancies were reevaluated by two other reviewers. The following data were extracted from each study: study design, sample size, country, patient characteristics such as age, gender, comorbidities, and past medical history when available, methodology of COVID-19 diagnosis, ophthalmic diagnosis, intraocular pressure when characterized, interventions, and treatments.

Results

A total of 31 papers were included in the final analysis. The study characteristics are summarized in Table 1. Most publications were case reports (n=27), with single entries included from a case series, case study, cross-sectional, and cohort cross-sectional. Many large-scale studies were excluded due to presence of preexisting ocular conditions or patients under the age of 18. A total of 816 patients were reported among the study participants, with 427 (52.3%) patients reported as male and 389 (47.7%) as female. Based on the characteristics of available data, there were no discernable associations between age or gender and ocular manifestations on presentation. The mean age was 42.18 years old with the ages ranging from 19 to 91 years old [1, 2, 29]. The study by Russe-Russe et al. had no age documented for their patient and thus was excluded from calculation of the average age in Table 1 [6].

**Table 1:** Patients’ characteristics

Mean documented age of patients	42.18 years
Documented age range of patients (patients under 18 excluded. No patients were 18)	19 - 91 years old
Male patients	427 (52.3%)
Female patients	389 (47.7%)

Geographic distribution: Most reports (n=8) originated in India, followed by the United States (n=6). Egypt and Turkey represented the largest patient populations with 426 and 360 patients included in their studies, respectively. Countries with one paper and one patient each: Brazil, China, Columbia, Israel, Japan, Morocco, Palestine, Spain, Qatar, Tunisia, United Kingdom, Venezuela (Table 2).

**Table 2:** Papers by countries of origin (patients represented)

Country	Number of publications	Number of patients
India	8	8
United States	6	6
Egypt	2	426
Turkey	2	360
Iran	2	5

Diagnostic Methods

RT-PCR was the most common COVID-19 diagnostic method (n=19), followed by polymerase chain reaction (PCR) (n=9), and serology (n=5). Two studies reported initially negative RT-PCR samples but later confirmed a positive diagnosis via enzyme-linked immunosorbent assay (ELISA) testing, so both methods were included [3, 30].

Disease Severity

Most patients included in this study were documented as being placed in the Intensive Care Unit (n=65) or generally admitted to the hospital (n=8). The extent of some COVID-19 infections was not reported, so the status of the patient was reported instead (e.g., hospitalized or home quarantined). Many other papers did not report or grade the severity of COVID-19 and those patients were not included in Table 3.

**Table 3:** Severity of COVID documented

Intensive Care Unit (ICU)	65
Hospitalized	8
Mild (asymptomatic & home quarantined)	7
Severe	3
Critical	1

Reported Time of Ocular Manifestations

In relationship to COVID-19 infection, the reported time of ophthalmic manifestations varied between studies. Earliest reported manifestations were five weeks prior to positive COVID-19 test [27], and the latest reported manifestation was three months following their history of COVID-19 infection [25]. The most reported time of manifestation was approximately one to two weeks following COVID-19 onset or positive test (12 of 27 reported cases). Of note, five of the 27 reported cases reported COVID-19 diagnosis after evaluation or diagnosis of ocular complaints.

Ocular Manifestations

Presenting symptom terminology varied greatly between studies, so symptoms were aligned with the American Association of Ophthalmology’s (AAO) list of symptoms to help with categorization of symptoms [42].

Inflammation of the eye, via conjunctivitis, was the most commonly documented presenting symptom with 56 (49.1%) documentations out of the 114 recorded [1, 2]. Symptoms, such as eye irritation, redness, and conjunctival hyperemia, were characterized as conjunctivitis in the study by Öncül et al. [1]. Discharge from the eye (via increased secretions or lacrimation) was also documented in their findings and was the third most common presenting symptom documented in our review (Table 4). Symptoms that were mentioned once: colors look different, dark curtain in vision, dilated pupils, double vision, vision loss. 

**Table 4:** Presenting symptoms by American Association of Ophthalmology’s (AAO) associated term (multiple symptoms were documented in 6 papers)

Ocular signs and symptoms	Number of patients
Inflammation, general	56
Vision loss, general (bilateral / right / left)	6 / 1 / 8 (15 total)
Discharge from the eye	10
Blurriness (bilateral / right / left)	3 / 3 / 0 (6 total)
Light sensitivity	5
Headache behind eyes	3
Red eye	2
Swollen eye	2
Dark spots in vision	2
Distorted vision	2
Irritation	2
Pain in eye (bilateral / right / left)	1 / 0 / 1 (2 total)
Vision loss, peripheral (bilateral / right / left)	0 / 1 / 1 (2 total)

General vision loss (decreases in visual acuity and scotomas) was the second most common diagnosis and was documented 15 (13.2%) times. This vision loss was often temporary and 14 of 15 patients recovered to baseline vision [3,6-9,12,16,22,24,26-29]. The exception to this recovery was seen in the study done by Mabrouki et al., in which the patient experienced complete blindness due to optic demyelinating neuritis and optic atrophy. Of note, the COVID-19 infection was found incidentally after the patient presented with acute bilateral vision loss [30]. The patient’s past medical history included an undocumented splenectomy, diabetes mellitus, and hypothyroidism [30]. 

Diagnosis

Conjunctivitis was the most common clinical diagnosis provided (n=127). The study conducted by Wasfy et al. accounted for 111 documented cases of diagnosed conjunctivitis [2]. All cases were temporary and resolved within 10 days of combined treatment of antibiotic, steroid, and artificial tears. Retinal affection, in the form of cotton wool spots, was also seen in this study but was temporary and resolved within five weeks. The study also documented six patients diagnosed with orbital fungal cellulitis, each documented as having recovered from COVID-19 infection with at least one pre-existing risk factor, e.g., diabetes [2].

The study conducted by Öncül et al. reported subconjunctival hemorrhage in five patients and vitreous hemorrhage in one patient [1]. In the patient who experienced vitreous hemorrhage, they were diagnosed with diabetes mellitus eight years prior. The patient did not receive routine diabetic eye examinations for four months as COVID-19 interfered with treatment. This patient was on anticoagulant drug therapy during the treatment period and had a severe cough beginning three days before the hemorrhage started [1].

Many patient cases were classified into various diagnoses based on the etiology of the illness (Table 5). Singular cases that were not included in Table 5 consist of vitreous hemorrhage, placoid band at level of ganglion cell (GCL) and inner plexiform layer (IPL), pachychoroid pigment epitheliopathy, optic neuritis associated with myelin oligodendrocyte glycoprotein (MOG) antibody, anterior uveitis, non-granulomatous anterior uveitis, mydriatic pupils, cavernous sinus thrombosis with central retinal artery occlusion, central serous chorioretinopathy, central retinal vein occlusion (CRVO) with cystoid macular edema, hemi-retinal vein occlusion (HRVO), Vogt-Koyanagi-Harada disease, visual snow, unilateral glaucoma, submacular hemorrhage, Purtscher-like retinopathy, right superior ophthalmic vein (SOV) thrombosis with pulmonary embolism, papilledema secondary to cerebral venous thrombosis (CVT), hypofluorescence fovea surrounded by irregular hyperfluorescent defects, and central foveal thinning, hemi-CRVO with macular edema, exudates, cotton wool spots, and posterior segment petaloid lesions radiating from fovea. 

**Table 5:** Ocular diagnosis documented

Conjunctivitis	127
Neuro-retinal affection	9
Secondary fungal orbital cellulitis	6
Subconjunctival hemorrhage	5
Optic neuritis	4
Episcleritis	3
Macular neuropathy	3
Pachychoroid and pachy vessels associated with choroidal hyperpermeability w/o ocular inflammation	3
Bilateral localized serous retinal detachment with central serous chorioretinopathy (CSC)	3
Keratitis	2
Scotomas	2
Paracentral acute medial maculopathy	2

Treatment and Interventions

Common treatment was aimed at COVID-19 management through the use of antimicrobials (n=13) and steroids (n=16) [2,5,8-16,21,26-30]. Some therapies reported are more extensive due to more severe cases of COVID-19 infection, including mechanical ventilation [1]. No ocular surgeries were documented in any of the studies; the most direct ocular treatment was ophthalmically administered eye drops [2,5,13,27]. Most ocular complaints recorded resolved without treatment on follow-up visits. Documented measurements varied from paper to paper. Some commonly performed examinations were visual acuity test and fundus examinations, optical coherence tomography, fluorescein angiography, and magnetic resonance imaging (MRI)/computerized tomography (CT) imaging of brain and orbits. The commonly used treatments are listed in Table 6. Singly mentioned interventions include analgesic, antihypertensive, phototherapy, laxative, disseminated intravascular coagulation (DIC) treatment, multidisciplinary care, standard of care, immunoglobulins, oral non-steroidal anti-inflammatory drugs (NSAID), vitamin, and proton pump inhibitor (PPI). 

**Table 6:** Treatments and interventions

Treatment	Number of patients
Steroids	16 (6 with taper, 4 as steroid eye drops)
Antimicrobials	13 (including antibiotics, antiparasitic, and antiviral agents)
Respiratory support	5 (including supplemental oxygen, and bronchodilators)
Non-steroidal anti-inflammatory drugs (NSAID) eye drops	4
Anticoagulants	4
Immunosuppressives	2
Plasma Therapy	2
Vascular endothelial growth factor (VEGF) antagonist	2

Discussion

The initial reports concerning COVID-19 focused on respiratory manifestations. However, our research demonstrates there are ophthalmic manifestations as well. In this systematic review, we identified papers published on the topic of ocular manifestations of COVID-19 in order to provide healthcare professionals with an updated report. We hope this study aids with the rapid identification and treatment of ophthalmic manifestations associated with COVID-19 disease.

**Figure 2:**
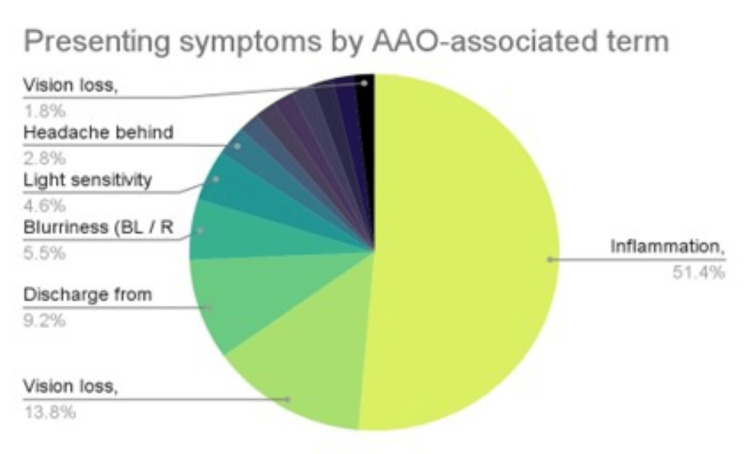
Presenting symptoms by American Association of Ophthalmology’s (AAO) associated term

The review revealed a total number of 109 ophthalmic presenting symptoms, with the most common being general inflammation of the eye (n=56), followed by vision loss (n=15), discharge (n=10), and blurriness (n=6) (Figure 2). Overall, the most reported diagnosis was conjunctivitis with the majority reported in the study conducted by Wasfy et al. who reported 111 of the 127 cases of conjunctivitis [2]. Interestingly, all six reported diagnoses of secondary fungal orbital cellulitis were also reported by Wasfy et al. [2].

Differences in the incidence and prevalence rates of conjunctivitis secondary to COVID-19 infection were seen in other studies. A meta-analysis by Loffredo et al. found the rate of conjunctivitis in COVID-19 positive patients to be 1.1% on average. Huang et al. reported that 27% (n=15) of their patients confirmed to be positive for COVID-19 experienced exacerbated ocular symptoms. Though they did not specify the exact pathology of symptoms they reported, this could suggest widely varying degrees of ocular manifestations in different populations. Nonetheless, the chances of some form of ocular symptoms persist, demanding attention to possible eye involvement in COVID-19 patients [44].

The time of onset of ocular manifestations in the context of COVID-19 illness is critical for healthcare professionals in consideration of care for infected patients. In our review, the time of ocular manifestations varied quite significantly and was not well documented between studies. The earliest manifestation was reported five weeks prior to a positive COVID-19 test and the latest was reported three months following a positive COVID-19 test. Although there are other causes of ocular pathologies, varying from viral to autoimmune, we are limited by the lack of disclosure of further investigation of other possible causes of ocular pathologies. Regardless, this gap of knowledge will open avenues for other future studies. Average time of presentation varied, with the most common timeframe being one to two weeks following a positive COVID-19 diagnosis, as reported for 12 out of 27 patients. This variability in the timeline of onset of ocular manifestations may be attributed to the methods of data collection which relied primarily on self-reporting of ocular manifestations by patients. The variable geographical health mandates prompted by local governments to prevent transmission of COVID-19 may have negatively disrupted the ability of ophthalmologists to conduct eye exams and other elective clinical services, which may have biased an accurate timeline of the onset of ocular manifestations.

In our systematic review, we found that 65 patients were placed in the ICU and eight patients were admitted to the hospital during the time of diagnosis of ocular manifestations. The association between severe COVID-19 illness and the prevalence of ocular manifestations has also been discussed in the literature with previous studies yielding variable data. Loffredo et al. found the rate of conjunctivitis to be 3% in severe and 0.7% in non-severe COVID-19 patients [45]. Similarly, Guan et al. [46], who retrospectively analyzed clinical characteristics of 1,099 COVID-19 patients, and Wu et al. [47], who retrospectively investigated the ocular characteristics of 38 COVID-19 patients, reported an increased incidence of conjunctivitis in patients with severe disease. In another study conducted by Layikh et al., conjunctivitis was significantly associated with severe COVID-19 illness [48]. However, Xia et al. did not find an increased incidence of conjunctivitis in severe COVID-19 in their prospective study of 30 COVID-19 patients that assessed the presence of the virus in tears [49].

Our findings add to the broader discussion of the association of conjunctivitis as a presenting symptom in severe COVID-19 illness. However, our study was limited by the fact that most manuscripts we reviewed did not document the severity of COVID-19 illness when documenting ocular manifestations, thus preventing us from studying a bigger sample size. Further studies with an emphasis on COVID-19 disease staging are necessary to better elucidate a potential association between the prevalence of ocular manifestations and COVID-19 severity.

Interestingly, Mabrouki et al. reported a case of complete blindness due to optic demyelinating neuritis and atrophy, but it is important to note the patient presented with acute bilateral vision loss before a confirmatory COVID-19 test was performed [30]. Though it is unclear if the patient’s COVID-19 illness was the causative factor of the acute bilateral vision loss, the poor prognosis may suggest that COVID-19 could exacerbate symptoms in previously affected eyes.

**Figure 3:**
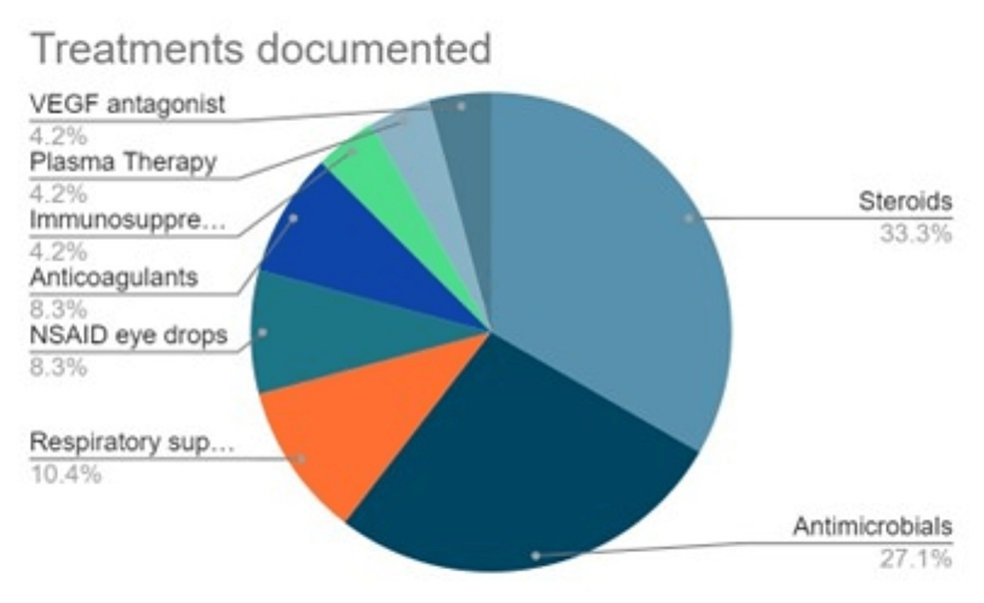
Treatments and interventions

It should be noted that most of the papers reviewed in this study did not discuss treatment of the ocular manifestations, and diagnoses were assumed to be self-resolving unless otherwise reported. The most common treatments were reported to be steroid use (n=16) and antimicrobial use (n=13) (Figure 3). The self-resolving nature of symptoms may suggest some association between ocular manifestations and active COVID-19 infection.

Limitations

Limitations of this study include the inability to control for potential medication-induced side effects, varying timelines in presentation post COVID-19 infection, and variations in workup of optic complications. Due to quarantine mandates and safety guidelines, patients may have opted to postpone doctor appointments that may have affected documentation of findings. Similarly, postponement of appointments may have exacerbated symptoms due to delayed treatments. Future studies that examine whether pre-existing ocular pathologies influence the severity and duration of COVID-19 induced ocular manifestations would provide further understanding of presentations.

Another limitation includes lack of reported severity of COVID-19, either not mentioned or use of nonstandard reports (Table 3). Lastly, ophthalmic examination is often not feasible in ICU patients.

Lastly, there is potential for publication bias as our literature search utilized PubMed exclusively. However, the potential publication bias is offset by the importance of the topic for general providers' educational purposes surrounding COVID-19. Additionally, limiting our search allowed for filtering of false information from unreliable sources, which is particularly important for COVID-19 information.

## Conclusions

Since the pandemic, it has been increasingly important to understand the signs and symptoms of COVID-19. Our results suggest that ocular manifestations of COVID-19 are extensive and can be the presenting symptom of infection, which may be beneficial in timely detection. Therefore, understanding how COVID-19 can present in the eye can aid in its diagnosis and treatment, potentially reducing long-term complications.
